# The Role of Verbal Fluency in the Cerebellar Cognitive Affective Syndrome Scale in Friedreich Ataxia

**DOI:** 10.1007/s12311-024-01694-x

**Published:** 2024-04-20

**Authors:** Louise A. Corben, Eliza Blomfield, Geneieve Tai, Hiba Bilal, Ian H. Harding, Nellie Georgiou-Karistianis, Martin B. Delatycki, Adam P. Vogel

**Affiliations:** 1 Bruce Lefroy Centre for Genetic Health Research, Murdoch Children’s Research Institute, Parkville, Victoria Australia; 2 Department of Paediatrics, University of Melbourne, Parkville, Victoria Australia; 3 Turner Institute for Brain and Mental Health, School of Psychological Sciences, Monash University, Clayton, Victoria Australia; 4 QIMR Berghofer Medical Research Institute, Brisbane, Queensland Australia; 5 Department of Neuroscience, Central Clinical School, Monash University, Melbourne, Australia; 6 Victorian Clinical Genetics Service, Parkville, Victoria Australia; 7 Centre for Neuroscience of Speech, University of Melbourne, Victoria, Australia; 8 Redenlab, Melbourne, Victoria Australia

**Keywords:** Friedreich ataxia, Cognition, Dysarthria, Cerebellum, Speech

## Abstract

Cerebellar pathology engenders the disturbance of movement that characterizes Friedreich ataxia (FRDA), yet the impact of cerebellar pathology on cognition in FRDA remains unclear. Numerous studies have unequivocally demonstrated the role of the cerebellar pathology in disturbed cognitive, language and affective regulation, referred to as Cerebellar Cognitive Affective Syndrome (CCAS), and quantified by the CCAS-Scale (CCAS-S). The presence of dysarthria in many individuals with ataxia, particularly FRDA, may confound results on some items of the CCAS-S resulting in false-positive scores. This study explored the relationship between performance on the CCAS-S and clinical metrics of disease severity in 57 adults with FRDA. In addition, this study explored the relationship between measures of intelligibility and naturalness of speech and scores on the CCAS-S in a subgroup of 39 individuals with FRDA. We demonstrated a significant relationship between clinical metrics and performance on the CCAS-S. In addition, we confirmed the items that returned the greatest rate of failure were based on Verbal Fluency Tasks, revealing a significant relationship between these items and measures of speech. Measures of speech explained over half of the variance in the CCAS-S score suggesting the role of dysarthria in the performance on the CCAS-S is not clear. Further work is required prior to adopting the CCAS-S as a cognitive screening tool for individuals with FRDA.

## Introduction

The neurological signs of Friedreich ataxia (FRDA) reflect the pervasive and complex neuropathology associated with this autosomal recessive condition. Progressive axial and appendicular ataxia are indicative of the cerebellar pathology particular atrophy of the Dentate Nucleus (DN) that typifies this disease [1]. Weakness, absent lower limb reflexes, impaired vibration sense and proprioception reflect associated degeneration of dorsal root ganglion, posterior columns of the spinal cord, dorsal spinocerebellar and corticospinal tracts and peripheral nerves [2]. Non-neurological signs such as diabetes, hearing and visual loss, scoliosis, foot deformity, and cardiomyopathy contribute to further morbidity and mortality in this life-shortening condition. Typically, onset of symptoms occurs between 10–15 years with a progression to loss of ambulation about 15 years later. FRDA occurs in most affected individuals as a result of a homozygous expansion in the Guanine-Adenine-Adenine (GAA) trinucleotide repeat in the first intron of *FXN*, resulting in a reduction of expression of frataxin, a mitochondrial protein involved in iron sulphur protein production, storage and transport [2].

In most individuals with typical onset FRDA, dysarthria is a core symptom manifesting as slowed speech, poor breath control and subsequent impaired intelligibility and naturalness in speech [3]. Whilst the role of the cerebellum in movement is well documented, numerous studies have also unequivocally demonstrated the role of the cerebellum in cognitive, language and affective regulation [4]. Given the putative role of cerebellar pathology in FRDA, it follows that several studies have attempted to quantify cognitive impairment, however these studies have been impacted by small sample size,; varying measures of cognition and use of timed outcomes that may be confounded by movement slowing in people with FRDA [5]. Verbal Fluency Tests (VFTs) are a common measure of cognitive capacity in neurological populations. However, the presence of dysarthria in individuals with FRDA can confound performance on VFTs potentially leading to false positive results [5, 6]. It is unsurprising that consensus regarding the presence, nature and progression of cognitive impairment in FRDA continues to be a source of uncertainty for the research community [5].

In 1997, Schmahmann and Sherman described the Cerebellar Cognitive Affective or Schmahmann Syndrome (CCAS) characterised by executive deficits, impairments in visuo-spatial organisation and visuo-spatial memory, changes in personality, disinhibition or inappropriate behaviour and linguistic impairments, which are apparent in many of the acquired and inherited ataxias [7]. The defining features of CCAS are now well documented across a range of spinocerebellar ataxias (SCAs) [4] thanks to the validation of the CCAS scale (CCAS-S) as a measure of the linguistic, executive and visuo-spatial components of cognitive control in individuals with SCA [8].

The CCAS-S comprises 10 items (Semantic Fluency, Phonemic Fluency, Category Switching, Verbal Registration and Recall, Digit Span Forward, Digit Span Backward, Cube Draw or Copy, Similarities, Go No-Go and Affect), and has an overall maximum score of 120. Performance on each item is scored as a “pass” or “fail” according to pre-set criteria. Eight of the 10 CCAS-S items necessitate a verbal response and of these items Semantic Fluency, Phonemic Fluency and Category Switching are VFTs. Failure on one item is considered to indicate the possible presence of CCAS, failure on two items probable CCAS, and failure on three items definite CCAS [8].

Few studies have explored the efficacy of the CCAS-S in distinguishing cognitive impairment in FRDA [9–12]. Two studies from the same research group examined performance on the CCAS-S and clinical metrics in FRDA alone [9, 10]. The first study explored the relationship between clinical severity as measured by the Scale for the Assessment of Ataxia (SARA) and the CCAS-S score in 19 individuals with FRDA, whereas the second study examined the impact of clinical severity (SARA score) as well as clinical parameters such as age at disease onset and disease duration on the CCAS-S score in a larger cohort (*n* = 39). Both studies concluded CCAS was both apparent in individuals with FRDA and, the presence of CCAS could be predicted by measures of clinical severity [9, 10]. Two further studies included individuals with FRDA within SCA cohorts [11, 12]. Thieme and colleagues applied the German version of the CASS-S to 64 individuals with SCA (including 20 individuals with FRDA) and 64 matched control participants [11]. The VFT CCAS-S items (Semantic Fluency, Phonemic Fluency and Category Switching) were significant in separating the overall cohort of individuals with SCA (including individuals with FRDA) from control participants. In contrast to that observed in the studies in FRDA alone, in this study clinical severity did not correlate with the CCAS-S score in individuals with FRDA. Furthermore, despite clear group separation on the VFT items, performance between individuals with FRDA and controls on the overall CCAS-S was not significantly different, although a higher number of items failed, and a lower CCAS-S score was noted in individuals with FRDA. Interestingly, the performance of individuals with FRDA on the CCAS-S differed from that reported by Naeije and colleagues [9]. Thieme and colleagues concluded that whilst the CCAS-S may be of use in quantifying cognitive impairment in some of the SCAs (such as SCA3) utility for detection of cognitive impairment is less so in SCAs with differing underlying cerebellar/cerebral pathology such as SCA6 and FRDA [11]. In a further publication Thieme and colleagues also highlighted that performance on the CCAS-S is dependent on age and education, warning that lack of age/education referenced values may lead to false positive results [12]. The presence of dysarthria, while not addressed in these studies, may also be a contributor to false positive results. Indeed, the presence of dysarthria when utilising a tool that relies heavily on spoken responses like VFTs, may obfuscate interpretation of CCAS-S performance. In recognition of this issue researchers have attempted to control statistically for slowed articulation in VFTs when examining group differences in other SCAs, but not in FRDA [13]. Whilst the utility of having an ecologically valid and clinically relevant tool to quantify cognitive changes in FRDA remains, it is crucial to understand the drivers of performance and avoid false-positive results on the CCAS-S for individuals with FRDA [6].

Herein we aimed to 1) explore the relationship between performance on the CCAS-S and clinical metrics of disease severity in a cohort of individuals with FRDA; and importantly, 2) to explore the relationship between performance on the CCAS-S and measures of intelligibility and naturalness of speech in a subgroup of individuals with FRDA. We hypothesized there would be a relationship between performance on the CCAS-S and clinical metrics of disease severity and moreover, this would be influenced by the presence of dysarthria in individuals with FRDA.

## Method

Adults homozygous for a GAA expansion in intron 1 of the *FXN* gene were invited to complete the CCAS-S during their annual clinic review (Friedreich ataxia clinic, Melbourne, Australia) or in the context of related research projects (the Melbourne arm of the SpeechATAX project [14] and the Neuroinflammation in Friedreich ataxia study [15]). Drawing participants from three sources mitigated the risk of bias and ensured a representative cohort across the spectrum of FRDA. Demographic information (sex, date of birth, years of education) was collected from all participants. In addition, participants underwent clinical rating of disease severity by administration of the Friedreich Ataxia Rating Scale (FARS) [16]. The FARS comprises timed measurements of neurological function, a functional disability staging score and a measure of activities of daily living. The FARS is scored out of 159 with higher scores reflecting greater disease severity [16]. In addition, clinical parameters related to age at disease onset (AAO, the age at which the affected person and/or family members first noted symptoms related to FRDA), disease duration (DD, current age minus age at disease onset) and the GAA repeat size on the smaller (GAA1) and larger (GAA2) *FXN* allele were recorded.

A sub-group of the cohort also underwent speech assessment, in particular intelligibility and naturalness of speech rated using a previously reported 0–4 scale (0 = no impairment, 4 = severe impairment) [17, 18]. The speech item (#4) of the Scale for the Assessment and Rating of Ataxia (SARA) [19] was also recorded for this cohort. This item is scored from 0 (normal) to 6 (speech unintelligible). Thus, there were three measures of speech: Intelligibility score, Naturalness score and SARA speech item. Note these participants also underwent the FARS clinical rating scale and recording of clinical parameters (AAO, DD and GAA repeat size).

The study was approved by Monash Health Human Research and Ethics Committee (HREC 02114A), Monash University (MUHREC 2017–7810) and the University of Melbourne (UoMHREC 1339394). All participants provided informed, written consent in accordance with the Declaration of Helsinki.

### Statistical Analysis

Data was checked for normal distribution using the Shapiro-Wilk test for normality. For all participants, descriptive statistics reported overall performance on the CCAS-S and further quantified pass/fail on specific subitems. For Group 1, the relationship between clinical parameters and clinical severity (measured by the FARS) and performance on the overall CCAS-S, as well as individual CCAS-S items, was explored using either Pearson product-moment correlation coefficients (r) or Spearman rank correlation coefficients (rho). For Group 2, the relationship between measures of speech and performance on the overall CCAS-S as well as individual CCAS-S items was explored using either Pearson product-moment correlation coefficients (r) or Spearman rank correlation coefficients (rho). Finally, the contribution of the measures of speech to the overall CCAS-S score was explored by multiple linear regression. Analysis was completed with IBM SPSS Statistics V.29 software (IBM Corporation, Armonk, NY, USA).

## Results

Fifty-seven adults with FRDA (33 female, mean age 38.9 years) participated in this study (Group 1). From this cohort 39 individuals with FRDA (Group 2) underwent an additional speech protocol (see Table 1 for demographic details of both groups).

**Table 1:** Demographic characteristics of cohort (*n* = 57)

Group Characteristics	Total Group 1 (*n* = 57)			Sub-Group 2 (*n* = 39)		
Male	24			14		
Female	33			25		
	M	SD	R	M	SD	R
Age	38.9	12.2	18–63	43.3	9.9	20–60
Education (years)	13.9	2.3	10–19	14.3	2.4	11–19
Age at Disease Onset (years)	15.1	6.9	1–34	14.9	7.6	1–34
Disease Duration (years)	23.4	11.3	3.8–49.3	27.9	8.9	13.8–49
GAA1 GAA2	619.2 900.5	217 191.5	126–1298 182–1345	644.5 925.3	230.3 199.8	126–1298 182–1345
FARS Score	95.3	28.7	32–150	109.4	23.3	66.5–150
Total CCAS score	85.8	15.9	46–114	83.5	17.1	46–114
Total CCAS Errors	2.4	1.8	0–7	2.2	1.8	0–7

Legend: Age = age at time of testing, FARS score = Friedreich Ataxia Rating Scale score (/159), GAA1 = size of GAA repeats on the smaller allele, GAA2 = size of GAA repeats on larger allele, Disease Duration = time since age of onset to age at time of testing, Total CCAS score = total CCAS scale score (/120), Total errors = total number of errors made when completing CCAS scale

### Performance on the CCAS-S and Clinical Metrics of Disease Severity (Group 1)

**Fig. 1:**
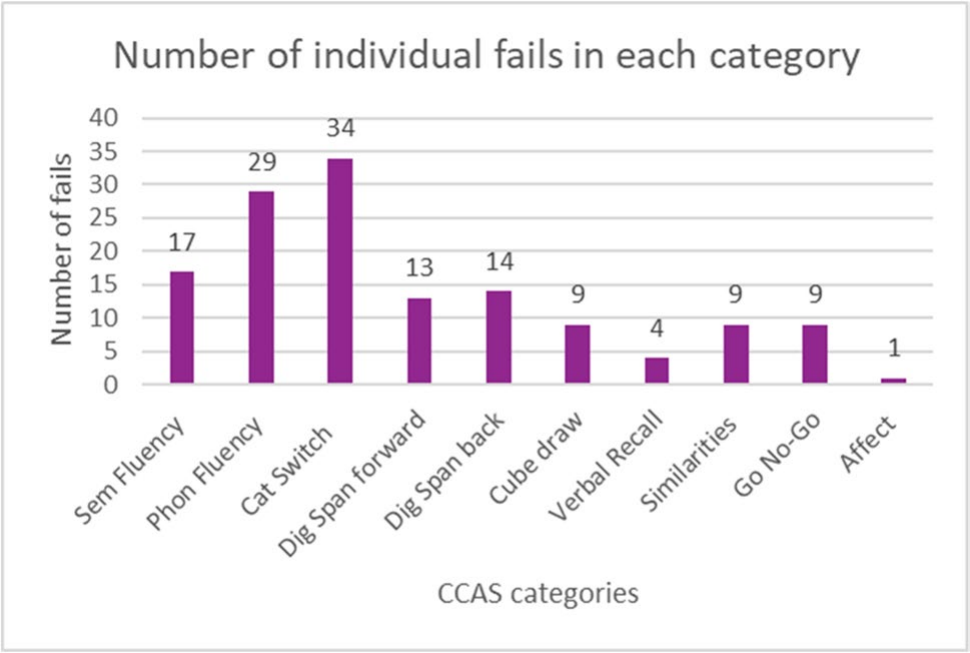
Number of individuals failing an item in each category of the CCAS scale. Legend: Sem Fluency = Semantic Fluency, Phon Fluency = Phonemic Fluency, Cat Switch = Category Switching, Dig Span Forward = Digit Span Forward, Dig Span Back = Digit Span Back

On average, participants in Group 1 scored 85.8 (/120) on the CCAS-S. Nine individuals were unable to complete the cube draw due to the severity of motor impairment and hence were scored out of 105. Failure on three tests (definite CCAS) was observed in 47% (27/57) of cases, failure on 2 tests (probable CCAS) was observed in 12% (7/57), failure on 1 test (possible CCAS) was observed in 28% (16/57) whereas 12% (7/57) of participants did not fail any test. Items that returned the greatest rate of failure were Category Switching (59%, 34/57 failed on this item); Phonemic Fluency (51%, 29/57 failed on this item) and Semantic Fluency (29%, 17/57 failed on this item). Only one participant failed the Affect item (see Fig. 1).

There was a significant correlation between the CCAS-S total score and the FARS score (r = 0.59, *p* < 0.001); AAO (r = 0.47, *p* < 0.001); GAA1 (r = 0.40, *p* = 0.002) and DD (r = 0.49, *p* < 0.001).

### Performance on the CCAS-S and Measures of Intelligibility and Naturalness of Speech (Group 2)

There was a significant correlation between the total score on the CCAS-S and SARA speech item (r = 0.72, *p* < 0.001), Intelligibility score (r = 0.61, *p* < 0.001) and Naturalness score (r = 0.60, *p* < 0.001). Examination of the relationship between the CCAS-S items returning the greatest rate of failure and measures of speech revealed significant correlations between performance on Category Switching and the SARA speech item (r = -0.54, *p* < 0.001), Intelligibility score (r = -0.58, *p* < 0.001) and Naturalness score (r = -0.50, *p* < 0.001). Performance on Phonemic Fluency demonstrated a significant relationship with the SARA speech item (r = -0.51, *p* < 0.001), Intelligibility score (r = -0.36, *p* = 0,02) and Naturalness score (r = -0.33, *p* = 0.04). Likewise, performance on Semantic Fluency was related to the SARA speech item (r = -0.61, *p* < 0.001), Intelligibility score (r = -0.41, *p* = 0.01) and Naturalness score (r = -0.42, *p* = 0.007). Whilst not returning a great number of errors, performance on the Verbal Recall item also demonstrated a weak relationship with the SARA speech item (r = -0.35, *p* = 0.02), Intelligibility score (r = -0.35, *p* = 0.02) and the Naturalness score (r = -0.36, *p* = 0.02).

Looking at the relationship between speech parameters and other items on the CCAS-S, there was a weak negative relationship between Digit Backwards and the Intelligibility Score (r = -0.32, *p* = 0.04). No further relationships were identified between any of the other CCAS-S items and the speech parameters.

### The Contribution of the Measures of Speech to the Overall CCAS-S Score

A multiple linear regression indicated Intelligibility score, Naturalness score and SARA speech item explained a significant amount of the variance in the CCAS-S score (F (3,36) = 13.81, *p* < 0.001). Moreover, *R2* indicates the measures of speech explains 54.9% of variance in the CCAS-S score (*R2* = 0.549, *R2*^*Adjusted*^ = 0.510). However only the SARA speech item (B = -11.89, t(39) = -3.30, *p* = 0.002) made a uniquely significant contribution to the model.

## Discussion

This study explored the relationship between performance on the CCAS-S and clinical metrics of disease severity in individuals with FRDA. In addition, this study explored the relationship between measures of intelligibility and naturalness of speech and scores on the CCAS-S in a subgroup of individuals with FRDA. We demonstrated a significant relationship between clinical metrics of disease severity including the FARS score, AOO, the repeat size on GAA1, DD and the CCAS-S total score. CCAS-S items that returned the greatest rate of failure were based on VFTs, that is Category Switching, Phonemic Fluency and Semantic Fluency. Unsurprisingly, there was significant relationship between these items and measures of speech. Furthermore, measures of speech explained over half of the variance in the CCAS-S score.

Based on the scoring criteria on the CCAS-S, the distribution of individuals in our cohort who displayed possible/probable/definite CCAS was 28/12/47%, with 13% of participants exhibiting no evidence of CCAS. This pattern is consistent with a previous study that reported a distribution of 35/12/25% with 25% of participants with FRDA (*n* = 20) not exhibiting CCAS [10], whilst Naeije and colleagues (*n* = 19) [9] reported a greater incidence of definite CCAS with a distribution of 21/15/65% and in a later study (*n* = 39) [10], 53% of individuals with FRDA demonstrated definite CCAS. Notably in our study, as with that of Thieme and colleagues [11], some participants with FRDA did not display any evidence of CCAS whereas in the studies by Naeije, Destrebecq and colleagues all participants failed at least one item [9, 10].

Closer examination of the items that contributed to a diagnosis of definite CCAS reveal those related to verbal fluency as prominent [11]. In our study, and consistent with other studies [9, 10], these items were Category Switching and Semantic/Phonemic Fluency, all of which are evaluated by timed measures of speech output. Given the profound impact of the dysarthria that typifies FRDA [3] it is difficult to ascertain if a lower score was related to an impairment in functions such as cognitive flexibility or abstract concept formation that underscore success in these items, or indeed are reflective of the dysarthria that typifies FRDA. Failure on these items may well reflect significantly slowed production of speech, rather than deficits in verbal fluency per se [5, 6]. This premise is supported by the strong correlations between verbal fluency items, measures of intelligibility and naturalness of speech and the SARA speech item and the regression analysis that confirmed the significant effect of speech on the total CCAS-S score. Failure on VFTs is not unique to FRDA and has also been reported in SCA3 and SCA6 [11], however apart from one study [13] failure on these items has largely been viewed as evidence of impaired executive function, rather than acknowledgement that these items comprise a timed speech response which may well impact the capacity to complete an item effectively and therefore result in a false positive result.

Limitations of this study include the small cohort of individuals (*n* = 39) who underwent detailed speech analysis in addition to administration of the CCAS. Future studies with larger cohorts are required to replicate and expand this exploration into the role of dysarthria in performance on the CCAS-S. Nevertheless, to our knowledge this is the largest study undertaken to explore the utility of the CCAS-S in quantifying cognitive function in FRDA. It is also the first study to examine factors such as dysarthria that have the potential to influence the CCAS-S scores in FRDA.

Understanding the cognitive profile of FRDA has long been challenging particularly due to the limitations of the current assessment tools available. A recent meta-analysis of cognitive studies in FRDA [5] confirmed the presence of mild cognitive changes in individuals with FRDA, however the nature and degree of impairment continues to be somewhat elusive. In this study we confirmed the relationship between CCAS-S and clinical metrics of disease severity in individuals with FRDA. It is understandable that in the need to identify a cognitive screening tool for FRDA, we may overlook the drivers of performance on cognitive assessment tools. To ensure we did not overlook such drivers of performance, we examined the impact of intelligibility and naturalness of speech on scores obtained on the CCAS-S. In so doing we were able to identify the presence of dysarthria as a significant driver of performance for individuals with FRDA completing the CCAS-S. Further work with larger cohorts and in the interim, caution is required prior to adopting the CCAS-S as a screening tool for individuals with FRDA. Specifically, studies involving larger FRDA specific cohorts and further exploration of the role of dysarthria in performance and comparison with control participants are required prior to confirming that the CCAS-S is a valid and effective cognitive screening tool for individuals with FRDA.

## Data Availability

The data that support the findings of this study are not openly available due to reasons of privacy however are available from the corresponding author upon reasonable request.
